# Smoking behaviour predicts tobacco control attitudes in a high smoking prevalence hospital: A cross-sectional study in a Portuguese teaching hospital prior to the national smoking ban

**DOI:** 10.1186/1471-2458-11-720

**Published:** 2011-09-23

**Authors:** Sofia B Ravara, Jose M Calheiros, Pedro Aguiar, Luis Taborda Barata

**Affiliations:** 1 Health Sciences Research Centre (CICS), Faculty of Health Sciences (FCS), University of Beira Interior (UBI), Av. Infante D. Henrique, 6200-506 Covilhã, Portugal; 2 Centro Hospitalar da Cova da Beira (CHCB), EPE, Quinta do Alvito, 6200-251 Covilhã, Portugal; 3 National School of Public Health, Universidade Nova de Lisboa, Av. Padre Cruz, 1600-560 Lisbon, Portugal

**Keywords:** Tobacco control, smoking, smoke-free policies, smoking ban, attitudes, healthcare professionals, hospital

## Abstract

**Background:**

Several studies have investigated attitudes to and compliance with smoking bans, but few have been conducted in healthcare settings and none in such a setting in Portugal. Portugal is of particular interest because the current ban is not in line with World Health Organization recommendations for a "100% smoke-free" policy. In November 2007, a Portuguese teaching-hospital surveyed smoking behaviour and tobacco control (TC) attitudes before the national ban came into force in January 2008.

**Methods:**

Questionnaire-based cross-sectional study, including all eligible staff. Sample: 52.9% of the 1, 112 staff; mean age 38.3 ± 9.9 years; 65.9% females. Smoking behaviour and TC attitudes and beliefs were the main outcomes. Bivariable analyses were conducted using chi-squared and MacNemar tests to compare categorical variables and Mann-Whitney tests to compare medians. Multilogistic regression (MLR) was performed to identify factors associated with smoking status and TC attitudes.

**Results:**

Smoking prevalence was 40.5% (95% CI: 33.6-47.4) in males, 23.5% (95% CI: 19.2-27.8) in females (p < 0.001); 43.2% in auxiliaries, 26.1% in nurses, 18.9% among physicians, and 34.7% among other non-health professionals (p = 0.024). The findings showed a very high level of agreement with smoking bans, even among smokers, despite the fact that 70.3% of the smokers smoked on the premises and 76% of staff reported being frequently exposed to second-hand smoke (SHS). In addition 42.8% reported that SHS was unpleasant and 28.3% admitted complaining. MLR showed that smoking behaviour was the most important predictor of TC attitudes.

**Conclusions:**

Smoking prevalence was high, especially among the lower socio-economic groups. The findings showed a very high level of support for smoking bans, despite the pro-smoking environment. Most staff reported passive behaviour, despite high SHS exposure. This and the high smoking prevalence may contribute to low compliance with the ban and low participation on smoking cessation activities. Smoking behaviour had greater influence in TC attitudes than health professionals' education. Our study is the first in Portugal to identify potential predictors of non-compliance with the partial smoking ban, further emphasising the need for a 100% smoke-free policy, effective enforcement and public health education to ensure compliance and promote social norm change.

## Background

Implementing a "100% smoke-free environment" policy, as proposed by the World Health Organization (WHO), is essential to reduce harm from smoking [1,2]. Hospitals should play an exemplary role in making smoke-free environments the social norm [3]. However, smoke-free policies (SFP) are not easy to implement and demand perseverance. Public acceptance and support, as well as strong administration commitment and effective enforcement are crucial to obtaining compliance [4-7]. In addition, support for and compliance with tobacco control policies can be influenced by the interaction of several psychosocial and cultural factors, such as socio-demographics, individual attitudes and beliefs, smoking behaviour and nicotine dependence, health beliefs and exposure to SHS, public health education campaigns, social and cultural norms concerning smoking, and tobacco industry marketing and regulation [8-16]. Although smoke-free hospital policies have been implemented in many European countries, exemptions and policy breaches remain frequent [3,17,18]. Hospital tobacco control policies and comprehensive smoking bans help reduce smoking prevalence [19-21] and policy breaches among staff [4,22]. Providing smoke-free environments has been proven not only to protect non-smokers, but also to encourage smokers to quit and participate in TC activities [23,24]. Recent research in countries with comprehensive SFP has shown that:

1. Hospitals, as workplaces, span the socioeconomic spectrum and provide case studies for studying smoking prevalence and behaviour in the adult population, the impact of tobacco control policies and compliance with smoke-free policies [25,26];

2. Evaluating smoke-free policy implementation in a hospital setting may serve as a guide to its sustainability in a broader national context [4];

3. Data is lacking concerning hospital staff smoking prevalence [25];

4. Smoking prevalence among health care providers (HCPs) remains high [27];

5. Implementing a total smoking ban in hospitals is particularly difficult and extending it to outdoor areas even more so [17,25];

Portugal has one of the lowest crude smoking prevalence rates in Europe (20.9%: 30.9% - males; 11.8%-females). It also has one of the highest never-smoking rates in Europe. Nevertheless, age-gender specific prevalence is high in young adults and the working population [28,29]. Since 1982, smoking has been banned on Portuguese healthcare premises. While some hospitals implemented smoke-free policies, enforcement tended to be lax and little change was observed in social norms [30,31]. Since 2008, a new smoking prevention law requires a complete smoking ban in all workplaces and on all non-mental healthcare premises [32]. There have now been several studies in a number of countries investigating attitudes to and compliance with bans on smoking in indoor public areas. But few have been conducted in healthcare settings and none to date in Portugal. Portugal is of particular interest because:

• current national smoking ban (NSB) is a partial one and is not in line with WHO recommendations for a "100% smoke-free" policy;

• timely public health education campaigns were not undertaken [33,34];

• awareness of SHS risks is low and effective smoke-free policy enforcement is lacking [28,35];

• little is known about the smoking behaviour and TC attitudes of Portuguese HCPs.

In October 2007, a Portuguese teaching hospital implemented a tobacco control programme before the national smoking ban came into force on 1 January 2008. Concurrently, staff smoking behaviour and attitudes were surveyed. The study was undertaken to assess among hospital staff:

• smoking prevalence and smoking behaviour

• tobacco control attitudes and beliefs

• factors associated with smoking behaviour and tobacco control attitudes and beliefs.

We also wanted to test if health care providers, especially those who should be role models and leaders on tobacco control, such as physicians and nurses [36], have the most positive attitudes to smoke free policies and tobacco control. In addition, the study also aimed to provide a baseline against which to measure attitudes and behaviour, and guide smoke-free policy sustainability, once the ban was implemented.

## Methods

### Setting

CHCB is a 340-bed university hospital, employing, according to the November 2007 payroll, 1, 112 salaried workers. The hospital serves a population of 65, 000 in a university city in central inland Portugal and is a major local employer.

### Internal tobacco control strategy

Sale of tobacco products was banned in the hospital and an information campaign about the new national policy was conducted three months before the national ban. Enforcement of the new hospital policy received institutional and public publicity through the hospital newsletter and local media. Concurrently, smoking cessation programmes were offered to employees and patients. A brief smoking prevention campaign was undertaken simultaneously. As part of the "National No Smoking Day" and "COPD Day", public information campaigns in the hospital's main lobby attracted professionals, patients, visitors and the media. All these activities took place between October and December 2007. The tobacco control strategy aimed to raise smoking prevention awareness and to promote smoke-free compliance among hospital staff, the patients and the wider community.

### Study design

An observational, questionnaire-based cross-sectional study was conducted of all salaried employees. A tailored version of the European Network of Tobacco-Free Health Care Services (ENSH) self-administered questionnaire was delivered to all department heads along with a request for cooperation in order to maximise staff compliance. The questionnaire included a cover letter explaining the study's aims and guaranteeing anonymity. Ethical approval for the study protocol and the survey implementation was obtained from the CHCB Hospital Research Ethics Committee. In order to ensure clarity and comprehensiveness of the questionnaire, the base questionnaire was piloted with a small group of 10 health workers (including participants of all the 5 subgroups of health workers) and revised according to their answers and comments. Questionnaires were returned to the smoking prevention department by internal mail, in sealed envelopes. The survey was conducted during November-December 2007.

### Sample

All salaried employees on the payroll in November 2007 were eligible for the study. Out of 1.112 sampled employees, 589 (52.9%) responded: 65.9% females; mean age 38.3 ± 9.9 years (min = 20; max = 68).

### Instrument survey, measures and definitions

#### Questionnaire

The original ENSH questionnaire was downloaded from the ENSH website, in October 2007. It was translated into Portuguese and then back into English to ensure accuracy. To ensure that all study objectives were met, in accordance with previously survey methodology [37] the original core questionnaire was supplemented with additional questions regarding smoking behaviour and "TC attitudes and beliefs". Information requested included demographics, personal smoking behaviour and smoking history and attitudes and beliefs concerning SHS and tobacco control policy. All data were self-reported. Information on smoking status, number of cigarettes smoked, smoking initiation age, intention to quit and previous attempts to quit was collected. The wording of questions and response options are shown in the additional file 1.

#### Smoking behaviour

The definitions used to describe smoking behavior are based on standard WHO definitions for tobacco use [38]. Respondents were classified as current smokers, ex-smokers and never-smokers. A current smoker is someone who at the time of the survey smokes any tobacco product either daily or occasionally and has smoked at least 100 cigarettes in his/her lifetime; an ex-smoker is someone who was a smoker but currently does not smoke at all, and reports having quit over than 6 months ago; an occasional smoker is someone who smokes but not every day; a non-smoker is someone who, at the time of the survey, did not smoke at all; a never-smoker is someone who has never smoked at all, or has smoked fewer than 100 cigarettes in his/her lifetime [38]. All answers regarding smoking behaviour were carefully reviewed to minimize misclassification. For bivariable analyses and logistic regression purposes, ex-smokers and never-smokers were treated as non-smokers. Nicotine dependence was evaluated on the basis of two items from "Fagerstrom's short-form test - "Heaviness of Smoking Index" (HSI). The sum of these two items is the HIS score, an objective measure of nicotine dependence [39].

#### TC attitudes and beliefs

Additional questions to address "TC attitudes and beliefs" were used to evaluate the following items:

1. Agreement with Hospital Smoke-Free Policy: HSFP (see additional file 1: questions 29-31).

2. Disagreement with SHS exposure in the hospital: SHSEH (see additional file 1: questions 32-34.1).

3. Attitudes and beliefs to the forthcoming national smoking ban: NSB (see additional file 1: questions 34.2-35.2).

Answers were assigned in a four-point scale and dichotomized (yes, no), depending on the content (see additional file 1). For data analysis, binary responses were coded as 1 (yes) and 0 (no). Answers on a four-point scale were aggregated and recoded in binary form as follows: strongly agree and agree (1); strongly disagree and disagree (0); always/almost always and often (1); sometimes and never/almost never (0). Each item regarding "TC attitudes and beliefs" comprised three questions. Answers were dichotomised as described above. A computed variable was created calculating the sum of the scores on each item. Values thus then varied between 0 and 3. These variables were then dichotomised as follows: 3 (1) and 0-2 (0). These three dichotomised variables represent the participants with the maximum score in all three items of each group versus the others. These were the main outcome measures of the bivariate and multivariate analysis, concerning "TC attitudes and beliefs".

#### Data Analyses

Data analysis was performed using SPSS, version 15. Frequency distributions were used to describe the data. Bivariable analyses were used to measure associations between selected variables, with statistical significance based on the chi-squared test for independence and Mann-Whitney tests to compare means or medians. The MacNemar test was used to compare categorical variables among matched samples. Two-sided tests of significance were based on the 0.05 level. Multilogistic regression (MLR) was performed to examine factors associated with smoking status, specifically: smokers versus non-smokers (MLR1). Independent explanatory variables included in the MLR1 model were those previously described in the literature as major determinants of smoking [28] and significantly associated with smoking behaviour in the bivariable analysis, namely: gender, age group, and professional group. Education and income were accessed indirectly according to occupation i.e. professional group. Non-binary variables, such as age group and professional group were transformed into suitable dichotomised versions. The professional group categories were: less educated and lower income (auxiliaries), versus others; age categories were: under and over 55. A second MLR analysis was performed (MLR2) to identify factors associated with stronger agreement with a smoke-free hospital policy, positive attitudes and beliefs regarding the national smoking ban and disagreement with SHS exposure in the hospital. The dependent variables were the three dichotomised variables which represent the participants with the maximum score in all three items of each group versus the others. These composite variables were described above, on the questionnaire section. Independent explanatory variables included in the MLR2 model were those previously described in the literature as potentially influencing SHS and TC attitudes and beliefs and significantly associated with at least one of the three items of "TC attitudes and beliefs" in the bivariable analysis. This included age, gender, smoking status and professional group ("role models" specifically: nurses and physicians, versus others). We also tested as a separated MLR2 model: all HCPs versus non-HCPs, physicians versus all others, and nurses versus all others. We included "role models" and HCPs in the model, in order to test if HCPs, especially physicians and nurses, have the most positive attitudes to smoke free policies and tobacco control. A final model was built using a backward stepwise procedure, starting with all variables found statistically significant in the previous step, and removing any non significant interaction. Hosmer and Lemeshow goodness-of-fit test was used at each step to assess the fit of the model. Results are presented as odds ratio and 95% confidence intervals (CI).

## Results

### Participants

Table 1 presents participants' socio-demographic descriptive characteristics, smoking status, and level of participation by professional group (PG). Age was re-classified into age groups, according to Table 2.

**Table 1:** Descriptive characteristics and smoking status of the sample and level of participation by professional group (PG)

PG	a)	b)	Males c)	Females c)	Participation d)	Smokers	Never Smokers	Ex- Smokers
Office staff	100	17.2	23.0	77.0	59.5	23.5	57.1	19.4

Physicians	37	6.3	67.6	32.4	30.8	18.9	59.5	21.6

Nurses	226	38.8	29.3	70.7	72.0	26.1	56.3	17.6

Other HCPs	46	7.9	21.7	78.3	46.9	21.7	54.3	23.9

Auxiliaries*	125	21.4	38.7	61.3	48.0	43.2	46.4	10.4

Other non-HCPs	49	8.4	53.1	46.9	50.5	34.7	46.9	18.4

Total**	583	100.0	34.1	65.9	52.9	29.5	53.4	17.1

a) Total number of responders per PG; b) % of the participants sample; c) % within the responders by PG; d) % within hospital staff by PG.

*In Portugal auxiliaries do not receive specific professional health education. In general they have less than 10 years' education (in this hospital 60% have less than 7 years' education; 40% ≤ 4 years). Nurses have a minimum of 15 years' education and physicians at least 18 years. Auxiliaries have the lowest income and physicians the highest. ** 6 missing

**Table 2:** Smoking behaviour by gender and age group (years)

Gender**	Age group	Smokers		Never-smokers		Ex-smokers		Total
		**n**	%*	**n**	%*	**n**	%*	

**Males**	20-34	39	50.0	38	48.7	1	1.3	78

	35-44	19	35.2	25	46.3	10	18.5	54

	45-54	16	37.2	10	23.3	17	39.5	43

	55-68	5	25.0	9	45.0	6	30.0	20

	Total	79	40.5	82	42.1	34	17.4	195

**Females**	20-34	29	18.2	105	66.0	25	15.7	159

	35-44	38	31.1	61	50.0	23	18.9	122

	45-54	21	29.2	40	55.6	11	15.3	72

	55-68	0	0.0	18	85.7	3	14.3	21

	Total	88	23.5	224	59.9	62	16.6	374

**Overall Smoking Prevalence**	Total 580	171	29.5	310	53.4	99	17.1	

*%within the age group

**4 missing data regarding gender & age group

### Smoking prevalence and smoking behaviour

Overall smoking prevalence was 29.5% (95% CI: 25.8-33.2). The majority of smokers reported being daily smokers (74.3%). Smoking behaviour was significantly associated with occupation (p = 0.024; Table 1). When compared with females, males had a significantly higher smoking prevalence (males: 40.5%, 95% CI: 33.6-47.4); (females: 23.5%, 95% CI: 19.2-27.8); p < 0.001) and smoked significantly more (median: 15 cigarettes/day for males; 10 cigarettes/day for females; p < 0.001). However, both genders reported starting smoking at the same age (median age: 18.0 years). Age gender-specific smoking rates are shown in Table 2.

Most smokers (76.2%; 112) reported low levels of nicotine dependence; 21.8% (32) reported moderate dependence and only 3 smokers (2%) reported high levels. MLR showed that being younger than 55 was the most important predictor for being a smoker (OR: 3.78; 95% CI: 1.43- 9.97; p = 0.007), followed by being a male (OR: 2.32; 95% CI: 1.58-3.40; p < 0.001) and by being an auxiliary (OR: 2.15; 95% CI: 1.40-3.31; p < 0.001). Most smokers (66.2%) reported that they wanted to quit and 81.0% that they were encouraged to do so by their colleagues. The majority of smokers (65.8%) had already tried, 32.0% admitted that they might need assistance with their next attempt, but only 20.0% admitted readiness to quit.

### Smoking in the hospital and TC behaviour

The great majority of smokers (70.3%) admitted smoking on hospital premises during working hours. The highest rates were reported by nurses (81.1%) and "other HCPs" (88.9%). Lower rates were found among administrative staff (40.9%) and physicians (42.9%). Besides occupation (p = 0.05), smoking in the hospital was associated with higher nicotine dependence (time of the first cigarette: p = 0.003; number of cigarettes per day: p < 0.001; HSI score: p = 0.007), and shift work (p = 0.002). Hospital staff reported smoking more often in the hospital (70.3%) than at home (43.7%) or in the car (53.8%); p < 0.001. Nicotine dependence was the only variable significantly related to smoking at home (p = 0.001) and in the car (p < 0.001).

### TC attitudes and beliefs

Table 3 presents attitudes and beliefs concerning TC. The great majority of the responders (97.6%) believed that SHS is harmful and agreed that the hospital should be smoke free (91.8%). Most of them believed that tobacco smoke is the principal indoor pollutant (97.3%) and agreed with the forthcoming national smoking ban (93.9%). The great majority of smokers believed that SHS is harmful (97.0%). Although to a lesser extent than the non-smokers, most smokers supported the smoking bans (HSFP: 85.4%; NSB: 85.8%; p < 0.001). Although 76.0% of the staff reported being frequently exposed to SHS in the hospital, only 42.8% reported that SHS was unpleasant for them, and only 28.3% complained about SHS exposure. Most of the hospital staff (74.6%) thought that the national smoking ban would help smokers quit, but only 25.7% admitted complaining about SHS in public places. The proportion of participants with maximum score on hospital smoke-free policy agreement was significantly higher than those reporting disagreement with SHS exposure in the hospital (p < 0.001). MLR2 analyses (Table 4) showed that attitudes to hospital smoke-free policies and to the national ban, and also disagreement with SHS hospital exposure, were related to smoking status, but not to gender or age. Physicians agreed less with hospital smoke-free policies but not to a statistically significant extent. Only one physician reported high levels of nicotine dependence. This physician reported less positive attitudes to tobacco control (TC) policies and less disagreement to SHS exposure. In addition, disagreement with SHS hospital exposure was significantly related to being "role models". MLR2 analyses showed that smoking status was the most important predictor of TC attitudes and beliefs. Being a smoker was associated with less positive attitudes to hospital smoke-free policy and to the national ban, and with less disagreement with SHS hospital exposure. We also observed that smokers with the highest nicotine dependence levels reported the lowest support for smoke-free policies and the less positive tobacco control attitudes (data not shown). However, their number is too small to use statistical tests. Being a physician or nurse was significantly associated with the strongest disagreement about SHS exposure in the hospital, but not with other indoor public places. Smokers who reported wanting to quit expressed greater hospital smoke-free policy acceptance compared with pre-contemplation smokers (p < 0.05), but did not report more positive attitudes to the national ban, neither stronger disagreement to SHS hospital exposure. Knowledge of SHS harmfulness was overwhelmingly reported and was not significantly related to age, gender, occupation, or to smoking status and nicotine dependence (data not shown).

**Table 3:** Tobacco Control attitudes and beliefs

**1**. **HSFP agreement**							
			**Smoking Status**				

			**Smokers**		**Non-Smokers**		**Statistics Tests**

**Agreement**	**N**	**%**	**n**	**%**	**N**	**%**	Chi^2 ^= 44.097

**Yes**	422	79.6	95	62.1	324	87.6	df = 1

**No**	108	20.4	58	37.9	46	12.4	p < 0.001

**Total**	530	100.0	153	100	370	100	

**2**. **Disagreement with SHS exposure in the hospital**							

			**Smoking Status**				

			**Smokers**		**Non-Smokers**		**Statistics Tests**

**Agreement**	**N**	**%**	**n**	**%**	**N**	**%**	Chi^2 ^= 32.017

**Yes**	144	26.1	15	9.5	127	33.0	df = 1

**No**	407	73.9	143	90.5	258	67.0	p < 0.001

**Total**	551	100.0	158	100	385	100	

**3) Attitudes and beliefs to the national smoking ban**							

			**Smoking Status**				

			**Smokers**		**Non-Smokers**		**Statistics Tests**

**Agreement**	**N**	**%**	**n**	**%**	**N**	**%**	Chi^2 ^= 10.035

**Yes**	91	18.9	14	10	75	22.5	df = 1

**No**	390	81.1	126	90	259	77.5	p = 0.002

**Total**	481	100	140	100	334	100	

**Agreement**: n and % of the participants with the maximum score in all three items of each group of "TC attitudes and beliefs", versus all others. **Yes: **participants who reported stronger agreement with a smoke-free hospital policy, more positive attitudes and beliefs regarding the national smoking ban and stronger disagreement with SHS exposure in the hospital. **No**: all the others.

**Table 4:** Tobacco Control attitudes and beliefs: MLR2

Dependent variable						
	**HSFP agreement**		**SHSHE**		**NSB**	

Independent Variable*	Wald p value	Odds ratio (95% C.I.)	Wald p value	Odds ratio (95% C.I.)	Wald p value	Odds ratio (95% C.I.)

Gender	0.19	0.73 (0.46-1.17)	0.81	0.95 (0.62-1.46)	0.59	1.15 (0.70-1.88)

Smoking status	< 0.001	4.29 (2.70- 6.79)	< 0.001	4.50 (2.52-8.04)	0.002	2.65 (1.43-4.92)

Professional group	0.46	0.84 (0.53-1.33)	0.01	1.65 (1.11-2.46)	0.58	1.14 (0.71-1.82)

Model explanation	79.9%	-	73.7%	-	81.1%	-

Hosmer and Lemeshow test	Chi value = 1.52; df = 5	p = 0.91	Chi value = 1.11; df = 5	p = 0.95	Chi value = 8.86; df = 6	p = 0.18

*Gender: male (1), female (0); Smoking status: non smoker (1), smoker (0);

Professional group: role models - nurses and physicians (1); all the others (0)

HSFP - Agreement with Hospital Smoke-Free Policy

SHSHE - Disagreement with Second Hand Smoke Hospital Exposure

NSB - attitudes and beliefs to the National Smoking Ban

## Discussion

Our study identified high smoking rates among hospital staff, especially among the less well educated and lower income groups. A strong occupation gradient was observed in smoking prevalence, the lowest rates being observed in physicians. This trend is consistent with other recent studies [25,26,40,41]. Smoking behaviour was related to age, gender, education, and income, in much the same way as occurs in the general Portuguese population [28,42]. The great majority of hospital staff, including smokers, supported and had positive attitudes to a hospital smoke-free policy and to the forthcoming smoking ban, as observed, in 2006, by the Eurobarometer survey [43]. Most smokers smoked on hospital premises and high exposure levels were reported. The majority of the staff did not report that SHS was unpleasant and only a minority complained about it, as observed in the general population [28]. Physicians' and nurses' TC behaviour and attitudes to hospital smoke-free policy and to the national ban did not correlate with their status as "role models". Being a smoker was the most important predictor of "TC attitudes and beliefs".

Smoking prevalence among hospital staff was high when compared with the general Portuguese population and even higher when compared with local population data [28]. In addition, smoking rates are higher than in countries where comprehensive TC policies have a longer history and effective enforcement [25,26,44-48]. However, when compared with other studies in southern Europe, overall smoking and female rates are lower and the crude male rate is higher [49-51]. This trend may be explained by the fact that Portugal is between phases 2 and 3 of the tobacco epidemic [42]. When compared with Italy and Spain, Portuguese females are less frequently smokers, while male rates started decreasing later than in those countries [42,52,53]. We must emphasise the high prevalence of non-daily smokers in our sample. According to Schiffman [54] non-daily smokers are becoming increasingly prevalent and more frequent among the younger population and females [55]. The lowest smoking rates were observed among physicians, in line with declining prevalence trends among physicians in Portugal [30] and other countries [56]. These trends [22,25,26,56,57], further illustrate the growing social gap in smoking prevalence and cigarette consumption [58]. As in other studies [59], smoking in the hospital was associated with higher nicotine dependence and shift work. Hospital staff perform their duties under stress and shift work overloads, and experience heavy emotional, social and physical demands [60]. This may partly explain why smoking in the hospital is related to shift work and occupation. As observed in other studies in southern Europe, female HCPs have a higher smoking prevalence rate than the overall female population. In some studies, female HCPs have higher smoking prevalence rates than males [49,51,61,62]. This may be associated with higher work stress levels and rapidly changing social roles [63], as well as tobacco industry strategies [64,65]. As in another recent study [25], nurses had the highest participation in the survey. This may indicate that nurses are more motivated to participate in smoking prevention programmes. Since nurses have a significant impact on smoking cessation among patients [66], team approach training programmes should always involve this group [67]. However, in yet other studies [25,61,62] nurses had high smoking prevalence rates. This may be a barrier to systematic proactive approaches to smoking cessation [68]. Physicians had the lowest collaboration in the survey. Thus, as responders are usually those most interested in the subject, we may assume that the non responders may have higher smoking rates and less positive attitudes. As in another study [4], physicians agreed less with the hospital smoke-free policy. In addition, despite reporting the lowest smoking rate among the HCPs, physicians' TC behaviour and attitudes to a smoke-free hospital and to the national ban did not correlate with their status as TC "role models". These findings illustrate the lack of interest and engagement of Portuguese hospital doctors in TC policies, as well as their limited understanding of its importance. This may contribute to the current lack of TC policies in Portugal, as has been clearly documented by Joossens and Raw [33,34]. We must also emphasise that physicians and nurses reported the highest levels of disagreement concerning SHS exposure in the hospital, but not for other indoor public places. These findings suggest that physicians' and nurses' normative beliefs concerning hospital TC policies differ than TC policies concerning other indoor settings. We conclude that smoking behaviour and Portuguese cultural norms and social beliefs influence support for and attitudes to TC policies more strongly than professional education and clinical experience. Given our study design and measurement limitations these results should be interpreted with caution. Our results nevertheless indicate that HCPs smoking prevalence remains high in Portugal. Our study also suggests that HCPs participants in this survey might not have received appropriate training in tobacco control. The high smoking prevalence, including smoking rates on hospitals premises and attitudes to SHS are not consistent with the public health burden of tobacco epidemics. As in another study [26], although most smokers wanted to quit and had already tried, only a few admitted that they might need help and very few admitted readiness to quit. Tailored smoking cessation programmes should be designed to raise HCPs' self-confidence and readiness to quit, and promote assisted quitting. Stress management programmes should be implemented to educate and promote effective stress management. Hospital-based health education and smoking cessation programmes should be gender-specific, as females are the main providers of practical patient care in hospitals and their smoking prevalence is high. These programmes should also address socio-economic status and nicotine dependence. Smoking prevention and tobacco control should be an essential component of undergraduate curricula in all health science schools. Tailored, gender-specific cessation programmes should be implemented in these settings. Tobacco control programmes in Portugal need to be more comprehensive, including recognition that smoking prevalence among young adults, and among working populations and HCPs remains worryingly high. Our findings also highlight the fact that Portugal, while smoking less than other European countries, mainly because women started smoking later, will move into a difficult tobacco control situation unless comprehensive tobacco control best practices are implemented. Future declines in tobacco use in Portugal will likely depend on the development of a comprehensive national tobacco control programme. Effective policies to target lower socio-economic groups, as well as workplace and university based smoking prevention programmes must also be top priorities of a national tobacco control strategy. Our study showed a very high level of acceptance of smoking bans, despite high smoking prevalence and the pro-smoking environment. Smokers reported significantly lower levels of disagreement over SHS exposure. These findings are consistent with previous research [4,11-15,25,59]. Strong support for smoke-free policies, even among smokers, has been reported in several countries that implemented smoking bans, even in the presence of pro-smoking social norms and high prevalence [11,69]. Even among smokers, support for and compliance with smoke-free policies increase over time, especially in countries that adopt strong policies while promoting public health education, self-regulation and social norm change [7,26,69]. Positive hospital staff attitudes to the forthcoming ban suggest that if such a ban is implemented rigorously the level of compliance will be high but, without a comprehensive ban and effective enforcement, attitudes alone are not sufficient to achieve a smoke-free environment. Moreover, most staff reported passive responses to high SHS exposure. As in other studies, this illustrates an important barrier to successful policy implementation since staff will not enforce the hospital smoke-free policy and will not challenge smokers on the premises [17]. This may be a potential predictor of non-compliance with the smoking ban, especially if public health education and effective enforcement are lacking [33-35]. Previous research has identified that where there is high smoking prevalence and a pro-smoking environment, most smokers fail to comply with smoking ban [12,35]. Moreover, compliance with smoking bans was not related to smokers' attitudes, but it is predicted by tobacco dependence and normative beliefs [12,14]. Portugal implemented a partial smoking ban, full of ambiguities and exemptions [32]. A partial ban does not fully protect all citizens from SHS exposure and has limited strength to promote a wide social norm change and may contribute to health and social inequalities. Most of the staff did not report being annoyed by SHS, despite high SHS exposure. Vardavas et al, 2011 [8], observed that non-smokers reported that they would proactively enforce the law, to promote compliance. Besides age and education, being bothered by SHS was associated with this intention. Therefore, a key strategy for policy-makers is to raise awareness of SHS health hazards while empowering non-smokers through public media campaigns and by informing them of their rights [8]. Another key strategy is to implement a strong smoke-free policy and monitor support and compliance over the time. Finally, hospital smoking bans "may create the paradox that smoking actually becomes more visible" [26], especially if smoking prevalence is high and smokers can be seen by the community smoking in shelters and around hospital entrances [25]. This will contribute to perceived high smoking prevalence and social acceptance. When HCPs are seen smoking, it clearly counters their status as role models and smoking cessation promoters, undermining social norm change. We therefore agree with others [26,70,71] who have argued that a comprehensive ban on smoking outside hospitals is a crucial component of good smoke-free hospital policy.

### Strengths and limitations

Our study is the first one in Portugal to focus on smoking behaviour and HCP attitudes to tobacco control prior to a national smoking ban. It was conducted in a high smoking prevalence hospital, with high SHS exposure levels. This study thus provides a baseline against which to measure smoking behaviour and attitudes now that the ban has been implemented. It also serves as a case study for the implementation of smoke-free policies in a pro-smoking environment. Given that our study focused on a single hospital, our conclusions cannot be extrapolated to Portuguese hospitals in general. And, while our study cannot be considered as representative of the national pattern, we nevertheless believe that our sample gives an indication of tobacco control standards in Portuguese hospitals prior to the implementation of the national ban. The cross-sectional design of our study leaves little scope for inferences as to causality. Also, since data were self-reported, social desirability bias cannot be excluded. The survey was, moreover, conducted just before the national ban was introduced; a period characterised by public debate and heightened awareness of SHS risks and the importance of smoke-free environments, reflecting a proximal effect of the introduction of smoke-free policies [72]. Furthermore, assessing smoking prevalence and attitudes using questionnaires may not reflect the actual situation. Sample selection bias and measurement errors may occur. Some reassurances as to accuracy are, however, provided by the fact that the ENSH questionnaire has been validated in many European countries and our additional questions validated by an earlier study. Since the response rate was above 50%, it is unlikely that our findings are biased by limited participation. Finally, the fact that few physicians participated in the survey means that this group is under represented.

## Conclusions

Our study identified high smoking rates among hospital staff, especially among the lower socio-economic groups. Most staff reported passive behaviour, despite high SHS exposure. This, plus the high smoking prevalence observed may contribute to low compliance with the smoking ban and low participation in smoking cessation activities [13,73]. Smoking behaviour had greater influence in tobacco control attitudes than health professionals' education. Smoking prevention and tobacco control should be top priorities in all health science schools and healthcare services. Our study is the first in Portugal to identify potential predictors of non-compliance with the partial smoking ban, further emphasising the need for a 100% smoke-free policy, effective enforcement and public health education to ensure compliance and promote social norm change. Our study also leads to the conclusion that post-ban evaluation and multi-centre studies should be undertaken in Portugal to monitor smoking behaviour, tobacco control attitudes and compliance with the smoking ban.

## List of abbreviations used

CI: confidence intervals; COPD: Chronic Obstructive Pulmonary Disease; ENSH: European Network of Tobacco-Free Health Care Services; HCPs: Health care providers; HIS: Heaviness of Smoking Index; HSFP: Hospital Smoke Free Policy; MLR: Multilogistic regression; NSB: national smoking ban; SFP: Smoke-Free Policies; SHS: Second-hand smoke; SHSEH: Second-hand smoke exposure in the hospital; TC: Tobacco Control; WHO: World Health Organization

## Competing interests

The authors declare that they have no competing interests.

## Authors' contributions

SBR and JMC designed the study and gathered the information, performed the statistical analysis, analysed and interpreted the data, and wrote the paper. PA performed the statistical analysis, analysed and interpreted the data, and critically reviewed the paper. LTB analysed and interpreted the data and critically reviewed the paper. All authors contributed, read and approved the final manuscript.

## Pre-publication history

The pre-publication history for this paper can be accessed here:

http://www.biomedcentral.com/1471-2458/11/720/prepub

## Supplementary Material

Additional file 1 **CHCB Staff- Tobacco survey Questionnaire- English version. Microsoft word**. This file contains the adapted version of the European Network of Tobacco-Free Health Care Services self-administered questionnaire. This version was administered on the survey. Click here for file
