# Supplementary material for: Smoking behaviour predicts tobacco control attitudes in a high smoking prevalence hospital: A cross-sectional study in a Portuguese teaching hospital prior to the national smoking ban
Source: BMC Public Health. 2011 Sep 23;11:720. doi: 10.1186/1471-2458-11-720 (PMC3189890; doi:10.1186/1471-2458-11-720)
Supplement: Additional file 1 — CHCB Staff- Tobacco survey Questionnaire- English version. Microsoft word. This file contains the adapted version of the European Network of Tobacco-Free Health Care Services self-administered questionnaire. This version was administered on the survey. [file 1471-2458-11-720-S1.DOC]

Additional file 1- CHCB Staff – Tobacco Survey Questionnaire

###

CHCB Staff – Tobacco Survey

As part of the “Smoke-Free CHCB Project” promoted by the administration, it was considered relevant to conduct an anonymous survey of staff smoking habits, opinions, attitudes and perspectives regarding the project, and then promote internal tobacco control strategies.

With this broad objective in mind we request your cooperation by completing the attached questionnaire which is totally anonymous and confidential. Your opinion and your participation are essential.

Thank you for your cooperation

Have a good day!

**Please fill in or put cross (X) in the appropriate box.**

**1.** **Gender**  M  F

**2. Age** ____ years

**3. Staff group**

 Administrative

 Physician

Nurse

Pharmacist

Auxiliary staff

Psychologist

Other allied health (please specify) ______________

Other (please specify) ______________

**4. Work schedule**  Regular daytime hours  Shift work

**5. Do you work during the night?**

 Never

 Occasionally

Always

**6. Did you smoke at least one cigarette during the last month?** Yes No

**7. Please select the appropriate statement that applies to you**

I have **never smoked**

I did smoke **a few cigarettes,** but never smoked regularly (**less** than 100 cigarettes in my entire life)

I smoke **regularly** (more than 100 cigarettes in my entire life) and **I smoke everyday**

I smoke **regularly** (more than 100 cigarettes in my entire life), but do not smoke everyday

I **used** to smoke **regularl**y (more than 100 cigarettes in my entire life), but **quit**

**8. If you have quit smoking please select the appropriate statement that applies to you**

I used to smoke regularly, but **quit** over the **last six months**

I used to smoke regularly, but quit more than six months ago but less than a year ago.

I used to smoke regularly, but **stopped more than a year ago**

**9. If you currently smoke please indicate**

**9.1** How many cigarettes do you smoke per day on average? _____

**9.2** At what age did you smoke your first cigarette? _____

**9.3** At what age did you start smoking daily? _____

**10. How soon after you wake up do you smoke your first cigarette?**

< 5 minutes  6-30 minutes  31-60 minutes   > 60 minutes

**11.1 Have you ever tried to stop smoking?** Yes No

**11.2** If yes, how many times have you tried? ____

**12. Please select the appropriate statement that applies to you**

I smoke, but would like to quit

I smoke, but would like to reduce

I smoke and do not want to reduce or quit

**13. Please select the appropriate statement that applies to you**

I smoke, but am considering quitting within a month

I smoke, but I am considering quitting in the next six months

I smoke and would like to quit, but am not considering doing so over the next six months

I smoke and do not want to quit

**14.** **Do you think you may need medical support to quit? Yes** **No**

**15.** **Do your colleagues encourage you to be a non-smoker / ex-smoker?** Yes No

**16. Please identify the situations that prompt you to smoke**

Meetings

Conflicts

Coffee Breaks

Treatment

Night work

Meals

**17. Do you usually smoke at work?** Yes No

**18. Please check the places where you usually smoke?**

Working areas

Office

Treatment areas

Lounge

Toilette

Cafeteria

Bar

Corridors

Stairs

Courtyards

Hospital entrance

**19.** **Do you usually smoke in the car?** Yes No

**20.** **Do you usually smoke inside your home?** Yes No

**21.** **Besides cigarettes, do you consume other tobacco products?** Yes No

**22. If yes, please check as appropriate**

Cigarillos

Cigars

Roll-your-own tobacco  Pipe

Other (please specify) ________________

**23.** **Does anyone smoke in your workplace?** Yes No

**24. Regarding the smoking habits of your partner, colleagues and close friends, please check the appropriate box.**

|  | ***smoker*** | ***ex-smoker*** | ***non-smoker*** | ***not applicable*** |
| --- | --- | --- | --- | --- |
| **Partner** |  |  |  |  |
| **Colleagues** |  |  |  |  |
| **Close friends** |  |  |  |  |

**25. Are there designated smoking rooms in the hospital?** Yes No

**26. Regarding smoking in the hospital. Please check the appropriate box.**

People smoke **only** in **smoking rooms**

People smoke in **other places** (please state) ____________________________

There is no smoking in the hospital.

**27. Do conflicts occur between smokers and non-smokers** Yes No

**28. In your department/service do you have designated areas for**

smokers Yes No

non-smokers  Yes No

 

**29. Do you agree that eliminating second-hand smoke to protect non-smokers should be further enforced?**

Yes No

**30.** **Do you agree that the current places where smoking is allowed should be revised?**

Yes No

**31. Do you agree that the hospital should be smoke free?**

Yes No

**32. Are you often exposed to second-hand smoke in the hospital?**

Yes No

**33. Is second-hand smoke exposure in the hospital unpleasant for you?**

I strongly agree  I agree  I disagree  I strongly disagree

**34. Do you complain when exposed to second-hand smoke? (please check the appropriate box).**

|  | Always/almost always | Often | sometimes | Never/almost never |
| --- | --- | --- | --- | --- |
| 34.1 Hospital |  |  |  |  |
| 34.2 Other indoor public spaces |  |  |  |  |

**35. Please state your opinion regarding the following statements**

|  | I strongly agree | Agree | I disagree | I strongly disagree |
| --- | --- | --- | --- | --- |
| 35.1 Smoking should be banned in all enclosed places |  |  |  |  |
| 35.2 A smoke- free policy in all enclosed places will contribute to help smokers quit |  |  |  |  |
| 35.3 Second-hand smoke is the major indoor pollutant |  |  |  |  |
| 35.4 Second-hand smoke endangers health |  |  |  |  |

Thank you
